# Near-Field Measurement of Six Degrees of Freedom Mining-Induced Tremors in Lower Silesian Copper Basin

**DOI:** 10.3390/s20236801

**Published:** 2020-11-28

**Authors:** Krzysztof Fuławka, Witold Pytel, Bogumiła Pałac-Walko

**Affiliations:** 1 KGHM Cuprum Ltd. Research & Development Centre, 2–8 Sikorskiego Street, 53-659 Wrocław, Poland; wpytel@cuprum.wroc.pl; 2 Faculty of Geoengineering, Mining and Geology, Wrocław University of Science and Technology, 15 Na Grobli st., 50-421 Wrocław, Poland; bogumila.palac-walko@pwr.edu.pl

**Keywords:** rotational seismology, mining-induced seismicity, prediction of rotational velocity

## Abstract

The impact of seismicity on structures is one of the key problems of civil engineering. According to recent knowledge, the reliable analysis should be based on both rotational and translational components of the seismic wave. To determine the six degrees of freedom (6-DoF) characteristic of mining-induced seismicity, two sets of seismic posts were installed in the Lower Silesian Copper Basin, Poland. Long-term continuous 6-DoF measurements were conducted with the use of the R-1 rotational seismometer and EP-300 translational seismometer. In result data collection, the waveforms generated by 39 high-energy seismic events were recorded. The characteristic of the rotational component of the seismic waves were described in terms of their amplitude and frequency characteristics and were compared with translational measurements. The analysis indicated that the characteristic of the rotational component of the seismic wave differs significantly in comparison to translational ones, both in terms of their amplitude and frequency distribution. Also, attenuation of rotational and translational components was qualitatively compared. Finally, the empirical formulas for seismic rotation prediction in the Lower Silesian Copper Basin were developed and validated.

## 1. Introduction

Measurements of rotational ground motions have recently been one of the most examined branches of seismology. Based on preliminary analyses, it is believed that the rotational component of the seismic wave may comprise valuable data for studying the physics of wave propagation [1,2,3]. In the past decade, many essential research works have been conducted in the field of identification and description of the rotational component of the seismic wave. As a result, the theoretical formulas describing ground rotations about three Cartesian axes on the ground surface were developed [4,5,6]. As it was mentioned in numerous research [3,7,8,9], rotational seismology has become an emerging topic for study in many fields of science. Increasing interest in the new field of geophysics translated into the rapid development of systems for measuring rotational components of ground motion. Some solutions, like TAPS rotational seismometer, were based on a pair of classical pendulum-based seismometers [10], while other systems were built from several commercially available geophones located in the circle line [11,12,13]. There is also a special type of sensors which enables to conduct direct rotational measurements. One of the first, and at the same time most commonly used, devices are liquid-based Rotational seismometers R-1 and R-2, manufactured by Eentec company [14,15]. These triaxial sensors allow to measure rotation with reasonable resolution and were used in many research fields [8,16,17]. 

Accessibility to sensitive rotation sensors turns into the possibility of conducting not only theoretical works but also practical research in the field of geophysics, mining, and earthquake engineering. In the beginning, most of the research works were related to measurement of strong-motion seismology in far-field [18,19,20,21,22,23]. This is due to the fact that records of earthquakes with high magnitude allow determining the rotation rate and the general characteristics of 6-DOF (six-degrees-of-freedom) motion. Then, information contained in the rotational component of the seismic wave was used for more specified purposes, such as seismic response analysis of complex structures like mining shafts [24,25,26], preliminary analyses of rotation rate in the regions of mining-induced seismicity [16,17,27], and physics of earthquakes as well [28,29]. Also, research works related to detecting gravitational waves generated by astronomical sources were conducted [30,31]. 

Currently, most of the research works are aimed at determining how the rotational component of the seismic wave affects the stability of structures [32,33,34]. Especially, measurements in the near-wave field seem to be most desired, since the characteristic of rotational seismic wave propagation in close distance from tremors’ hypocenter was not fully investigated yet [35,36]. It may be expected that high-energy tremors with the source located in close vicinity of such objects like shaft or tailing ponds may significantly affect their stability. In recent works, it was highlighted many times that from an engineering point of view, seismic rotation may cause visible damage in high-rise buildings and long horizontal construction such as pipelines or dams [37,38]. As it was pointed out in recent research works when analyzing the stability of long structures or objects with great volume, i.e., dams of flotation tailing ponds, both Spatial Variation of Earthquake Ground Motion and the rotational component of seismic wave play a significant role in overall seismic load characteristics [39,40,41]. Still, vast research works are based on rotational data generated from earthquakes in the far-field. As a result, in most cases, recorded values of rotation do not exceed the level of mrad^−1^.

According to recent research, near-field measurement of strong-motion data may contribute to formulating additional seismic loads on high structures in terms of the rotational seismic excitations. In References [42,43], it was proven that the rotational component of ground motion may significantly affect the level of seismic response of the mining shaft. Also, new formulations of seismic intensity for the multi-story buildings with the use of rotational ground motion data were developed [32,34]. Still, there are many aspects related to rotational ground motions, which should be investigated. Such parameters like frequency characteristic, amplitude distribution in near-wave field, and damping factors of the rotational seismic component may contribute to a better description of dynamic seismic load and its influence on engineering constructions. Especially on the areas of mining-induced seismicity, where the frequency of tremors’ occurrence may reach even a few hundred per year. 

**Figure 1:**
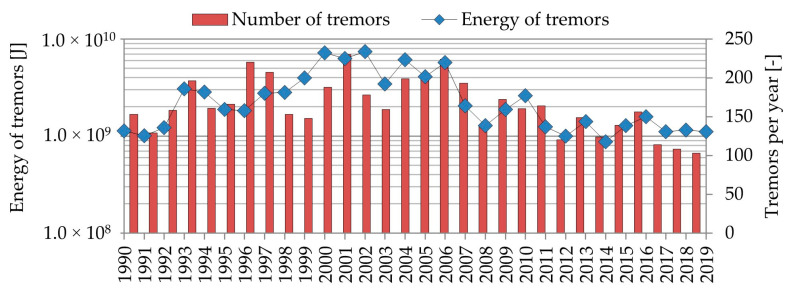
High-energy tremors recorded in the Lower Silesian Copper Basin (LSCB) region since 1991.

One of mining regions significantly affected by induced seismicity is the Lower Silesian Copper Basin (LSCB), which is located in Lower Silesian voivodeship, Poland. In this area, underground copper excavation has been continuously conducted over the last 60 years. As a result, an area of over 800 km^2^ has been affected by negative consequences of underground exploitation such as surface settlement or seismicity with relatively high intensity. Subsequently, there are hundreds of high-energy mining-induced tremors observed in the LSCB area each year (Figure 1). High intensity of seismic activity destructively affects closely located buildings and engineering infrastructure. Each year, significant damages of buildings are reported in the LSCB region [44]. Damages are present mostly in the form of visible cracks of walls and roofs. According to regular reports concerning the seismic intensity, in many cases, seismic load calculated based on parameters of translational seismic waves should not generate visible damages in building structures. So, it may be expected that currently, seismic load may be underestimated due to the omission of three rotational components of seismic waves in conducted analyses. 

**Figure 2:**
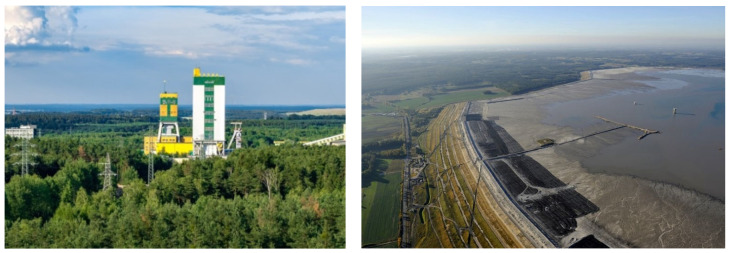
**Left**—Rudna-I Shaft of the Rudna copper mine, and **right**—the slope of Zelazny Most storage facility.

Still, the effect of seismic excitations is far more significant in the case of high structures like multistory buildings and mining shafts, which may amplify the level of the vibration at the highest part of the object [3]. At the moment, there are over 30 shafts in the LSCB. Their height varies from several to even 70 m (Figure 2—left). Most of them are used as ventilation shafts. Eventual loss of stability or even visible damage of shaft structure may generate a huge threat for people working both on the surface and in underground excavations. Besides shafts and residential buildings, there is one more object in the LSCB area to which special attention should be paid. Namely, in the area affected by seismic activity, Zelazny Most—the biggest flotation tailing pond all over the world is located (Figure 2—right) [45]. 

The surface of the facility at the moment is close to 16 km^2^, but in the next few years, it will exceed 22 km^2^. This is due to the necessity to ensure its operational capabilities until the year 2048 [46,47]. After the expansion stage, the capacity of the object will reach over 965 milions m^3^. The potential failure of one of the dams may cause hundreds of deaths in villages located in close distance from the object. Also, the ecological disaster should be expected as well. To ensure that the Zelazny Most facility is safe enough, a detailed risk assessment is conducted before any development of the facility. Numerical analyses combined with a well-developed monitoring system and comprehensive hazard evaluation methods generally make the object one of the best managed in terms of safety procedures. Nevertheless, it has to be highlighted that in currently used methods of slope stability calculations, only the translational component of the dynamic load is considered. In the literature, there are no attempts of implementation of rotational load into calculations, which is related to a lack of rotation measurement on such objects like dams and mining-waste storage facilities. 

Within this paper, the outcomes of preliminary seismic rotation measurement in the LSCB area will be presented. The results of near-field measurement conducted near the Rudna-I mining shaft and on the slope of the Zelazny Most tailing pond will be analyzed in terms of their amplitude and frequency distribution. Based on gathered data, attempts of predictive rotation formulas determination will be conducted. It is worth mentioning that the analysis presented in this article is based only on high-energy mining tremors located at a low distance from the source. This is a visible novelty and added value to previous studies where the analyzed tremors were located at a large distance or were characterized by low energy.

## 2. Materials and Methods

**Figure 3:**
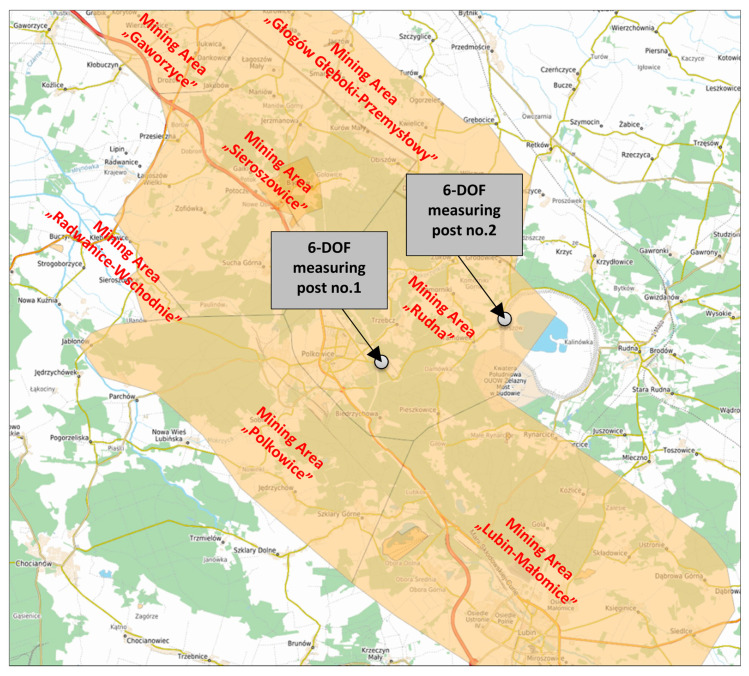
Location of six-degrees-of-freedom (6-DOF) measuring posts in the LSCB area.

The preliminary 6-DOF measurements in the LSCB were conducted in the Rudna mining area, which is recently characterized by high seismicity (Figure 3). 

**Figure 4:**
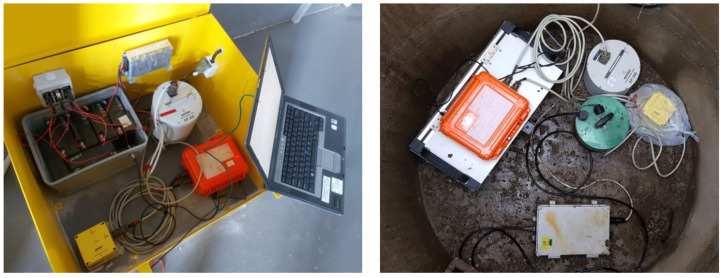
Systems for 6-DoF measurement installed near the Rudna-I shaft (**left**) and at the Zelazny Most tailing pond (**right**).

The first measuring post was installed at the concrete base near the Rudna-I shaft in order to determine the construction response for rotational load generated by the tremor in the near-wave field (Figure 4—left). The second station was mounted in a 2 m-deep concrete well, located at the dam of the Zelazny Most tailing pond (Figure 4—right). 

*Y*-axes of all seismometers were turned to the North direction. Rotational seismometers, due to the small weight, were grouted to the floor, to ensure proper measurement.

### 2.1. Seismic System for 6-DoF Measurements

All seismometers used during the field measurements, as well as recorders, were manufactured by Eentec company, Kirkwood, MO, US. In both measuring posts the same type of seismometers was utilized. Namely, the rotational motion was recorded with the use of triaxial rotational seismometer R-1, which utilizes electrochemical technology and is characterized by high measurement sensitivity. According to the manufacturer, this type of sensor enables to measure the rotational velocity of the wave while ensuring relative insensitivity to translational movement. In turn, to determine the level of translational vibrations, the EP-300 seismometer was utilized. Due to its low energy consumption, EP-300 is intended for both stationary and field applications. Efficient electrodynamic sensors provide a wide dynamic range, high stability, and linearity throughout the entire recording bandwidth. The measurement of the vibration velocity is carried out by three identical electrochemical sensors installed perpendicularly along the *X*-, *Y*- (horizontal components), and *Z*-axis (vertical component). Basic parameters of the used seismometers are presented in Table 1.

Vibrations were measured and recorded continuously. For this purpose, two 24-bit seismic recorders DR-4050P were utilized. Removable 32 GB USB memory allows for conducting 1-month continuous measurements, with a sampling rate of 500 Hz. Time was synchronized with the use of Global Positioning System (GPS) antennas. 

### 2.2. Data Processing

Both seismic posts were located near the area where other technological processes were continuously conducted. Due to close proximity to the seismic source, recorded waveforms were significantly contaminated by high frequencies, which exceed the value of the upper bandwidth of both types of seismometers. Therefore, signals before further processing were filtered with the use of bandpass filters, which allow extracting only amplitudes transferred with the chosen frequency range [48]. Of course, an ideal filter would have a gain of 1 in the passband while all frequencies contained in the stopband would be cut off. Still, recent advances in signal filtering do not allow to meet these requirements. Therefore, during the filtering process, a transition region has been generated at the contact line of the passband and the stopband. In this region, the gain of the filter changes steadily from 1 in the passband to 0 in the stopband. The attenuation of signal values due to the filtering process may be evaluated with the following formula:

(1) $${\frac{A_{0}\left(f\right)}{A_{I}\left(f\right)}|}_{dB}=20Log_{10}\left(\frac{A_{0}\left(f\right)}{A_{I}\left(f\right)}\right)$$

To ensure the most reliable results, the waveforms were filtered in a frequency range close to the bandwidth of the seismometers. In the case of the R-1 seismometer, the bandpass frequency was set up as 1–20 Hz. In the case of a translational sensor, waveforms were filtered in the rage of 1–40 Hz to avoid electrical noise, which is observed near 50 Hz.

## 3. Results

**Figure 5:**
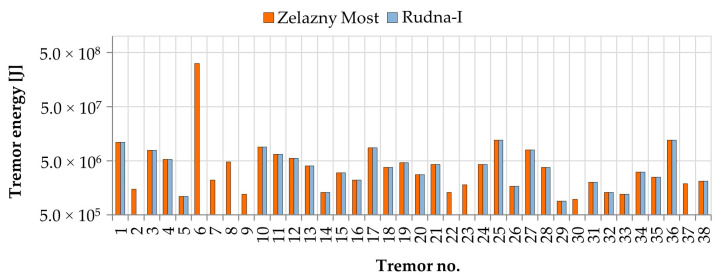
The energy of mining tremors recorder at a distance < 8 km.

During eleven months of measurements, hundreds of seismic tremors were recorded at both monitoring stations. According to current research, low-energy tremors (E < 10^6^ J) in most cases do not generate load which may destructively affect the object stability [49,50,51,52,53]. Therefore, for the purposes of further analysis, only high-energy events (E > 10^6^ J) located at a distance below 8 km from measuring posts were chosen. As a result, at the seismic post located at the dam of the Zelazny Most tailing pond, 38 high-energy tremors were recorded. In the case of the measuring station located near the Rudna-I shaft, some technical issues occurred due to the necessity of a battery supply upgrade. Thus, in this station, only 29 high-energy events were recorded (Figure 5). 

**Figure 6:**
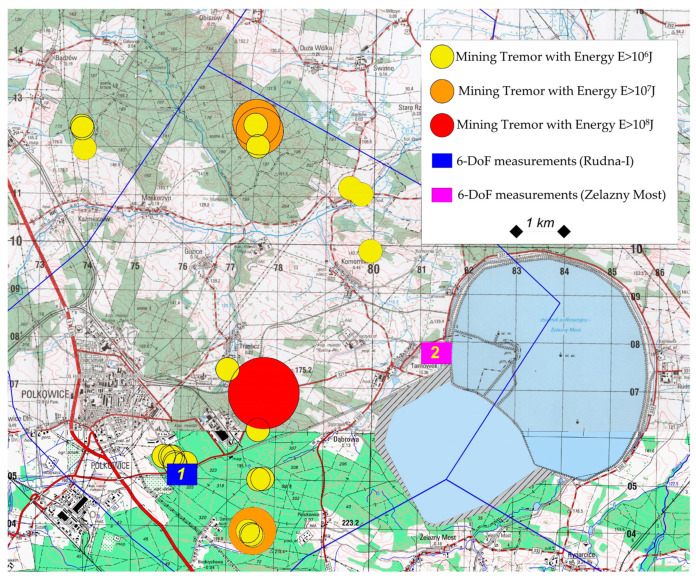
Epicentric location of recorded high-energy tremors.

The exact location of each high-energy seismic even is presented in Figure 6.

As one may conclude, most of the tremors have the source in close vicinity of the seismic station number 1 (Rudna-I). Moreover, seven tremors were observed just under the measuring site. In the case of the Zelazny Most tailing pond, most of the tremors were located at a relatively far distance, reaching up to 8 km. The closest tremor was located about 2.3 km from the measuring site. 

### 3.1. 6-DoF Amplitude Characteristic 

As it was already mentioned, in most seismic codes and mining intensity scales, the amplitude of seismic vibration is presented only in the translational domain. The rotation, due to a lack of reliable and accurate measurements, has been omitted. To determine what is the actual level of the rotational velocity of seismic wave generated by mining-induced tremors and how it correlates with translational motion, the analysis in time domain was conducted. Therefore, on the basis of the obtained seismic measurements about three perpendicular axes, the values of peak ground rotational velocity (*PG_RV_*) and peak ground velocity (*PG_V_*) were determined according to the following formula:

(2) $$PG_{V}{=}_{t}^{max}\sqrt{TV_{x}^{2}\left(t\right)+TV_{y}^{2}\left(t\right)+TV_{z}^{2}\left(t\right)}$$

where *PG_V_*—peak ground translational velocity at the measuring site, *TV_x_*—horizontal peak ground translational velocity in the East–West direction, *TV_y_*—horizontal peak ground translational velocity in the North–South direction, and *TV_z_*—vertical peak ground translational velocity.

(3) $$PG_{Rv}{=}_{t}^{max}\sqrt{RV_{x}^{2}\left(t\right)+RV_{y}^{2}\left(t\right)+RV_{z}^{2}\left(t\right)}$$

where *PG_RV_*—peak ground rotational velocity at the measuring site, *RV_x_*—horizontal peak ground rotational velocity in the East–West direction, *RV_y_*—horizontal peak ground rotational velocity in the North–South direction, and *RV_z_*—vertical peak ground rotational velocity. 

**Figure 7:**
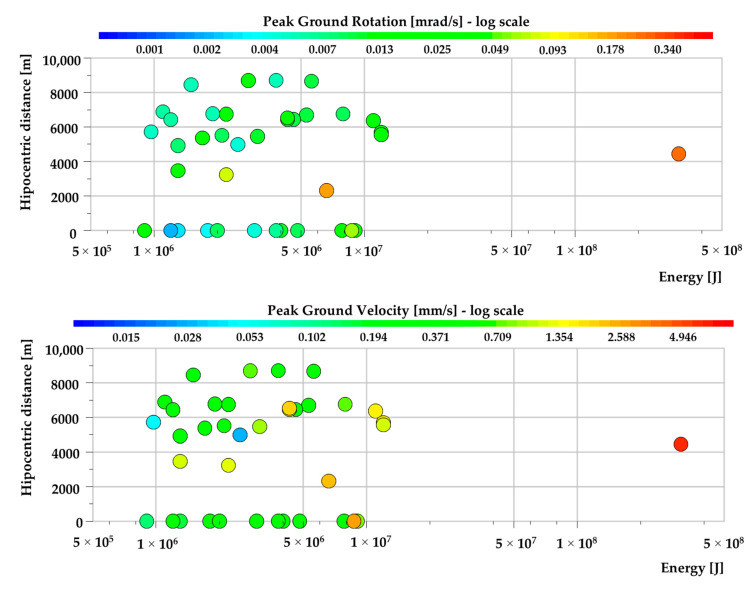
The values of and *PG_RV_* (**top**) *PG_V_* (**bottom**) recorded at the dam of the Zelazny Most tailing pond.

In Figure 7, the calculated values of maximum rotation and translation at the Zelazny Most tailing pond are presented. The color scale (*Z*-axis) is presented in the logarithmic domain to provide legible results. Some tremors were triggered during the group blasting works. As a result of the firing of subsequent faces in the group blasting, numerous seismic waves were generated. Total time of the whole blasting varies from 5 up to even 15 s. If a tremor occurs during the blasting works, it is extremely difficult to extract exact P-wave arrival time, because multiple amplitude peaks generated by detonation of explosives are present in the recorded waveform. In such case, determining the source is impossible or may be related with significant error. Therefore, the distance of such events from the measuring site was described as 0. Still, the peak amplitude of the seismic wave generated by a mining tremor is many times higher in comparison to peak amplitude generated by blasting works. As a result, a seismic event without a determined hypocentral location does not negatively affect the analysis of the peak amplitude characteristic.

One may conclude that in 85% of the analyzed cases, rotational velocity does not exceed the level of 0.1 mrad/s. The higher amplitudes of rotational vibration were observed only in the case of 6 tremors located at a distance below 4.5 km. When analyzing the amplitude distribution, it may be observed that distance of the measurement point from the seismic source may affect the amplitude level more that energy of the event. The maximum value of the peak ground rotational velocity was generated by a high-energy seismic tremor with E = 3.1 × 10^8^ J at the distance of 4.446 km from the source. The maximum *PG_RV_* was equal to 0.46 mrad/s. In case of translational motion, the amplitude distribution in relation to energy and distance from the event looks slightly different. Namely, PG_V_ is less sensitive to the rise of hypocentral distance while the main parameter affecting the value of amplitude was the energy of the tremor. 

**Figure 8:**
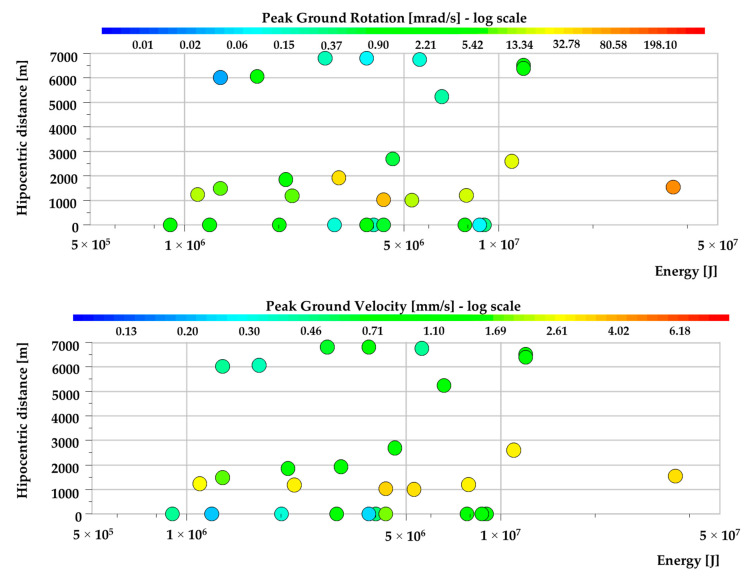
The values of and *PG_RV_* (**top**) *PG_V_* (**bottom**) recorded near the Rudna-I mining shaft.

The waveforms recorded at the second measuring post near Rudna-I bring valuable data due to very close proximity from the seismic source. According to the recorded dataset, high-energy tremors which occur at a distance below 2 km nearly in all cases were related to rotational velocity over 1 mrad/s (Figure 8). Moreover, the maximum rotational velocity of seismic wave reached the value of 195 mrad/s and was caused by a seismic tremor from 29 August 2019 with the energy of 3.6 × 10^7^ J located at a distance of 1550 m from the measuring post. At the same time, the records of transitional seismic motion in case of the strongest events were significantly distorted. After the filtering process, the condition of the obtained waveforms was visibly improved. Thus, the PG_V_ varied in the range of 0.01 up to 4 mm/s. Nevertheless, it needs to be highlighted that the real amplitude of translational velocity could be much higher than recorded ones. 

On the basis of the obtained *PG_RV_*, Peak Ground Rotation (*PG_R_*) was also calculated. For this purpose, the velocity records were integrated to rotation rate in mrad. To make the results more legible, mrad were also converted to degrees according to the formula:

(4) $$x^{{}^{\circ}}=x_{rad}\cdot \frac{180^{{}^{\circ}}}{\pi }=x_{rad}\cdot 57.29577951308$$

**Figure 9:**
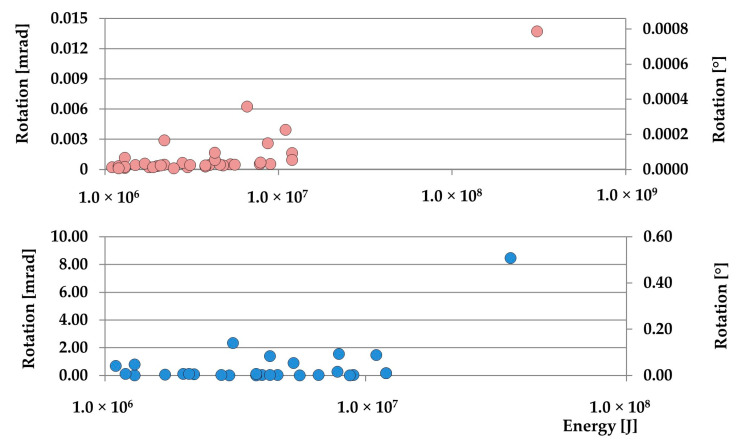
Calculated Peak Ground Rotation (*PG_R_*) at the slope of Zelazny Most (**top**), and near the base of the Runa-I shaft (**bottom**).

The results of the calculations are presented in Figure 9.

When analyzing the rotation rate, one may conclude that *PG_R_* observed near the Rudna-I shaft is about 103 times higher than at the dam of the Zelazny Most tailing pond. Maximum observed rotation in the first measuring post exceeded the value of 8 mrad (about 0.5°), while the rotation at the second station does not exceed the value of 0.014 mrad (about 0.00075°).

### 3.2. Dominant Frequency of Translational and Rotational Components of Seismic Wave

**Figure 10:**
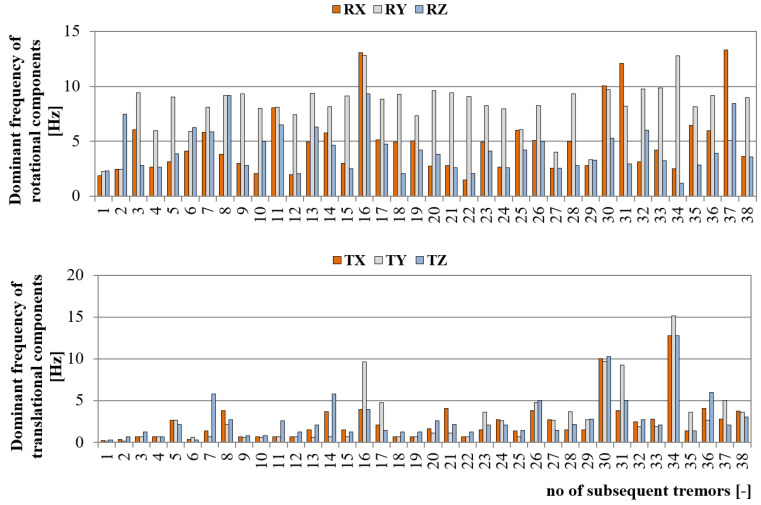
Dominant frequency of six components of seismic wave recorded at the slope of Zelazny Most.

**Figure 11:**
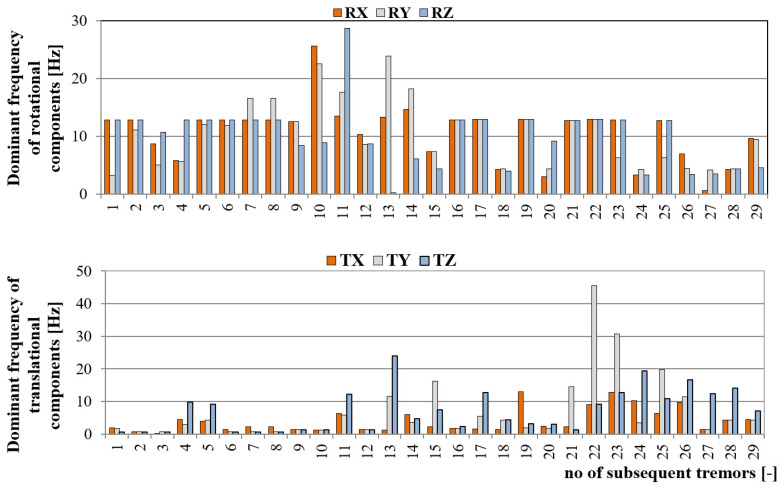
Dominant frequency of six components of seismic wave recorded near the Rudna-I shaft.

The second parameter which affects the dynamic load of engineering structures is the frequency content of the seismic wave. According to the authors’ experience, in the LSCB area, the dominant frequency of translational seismic vibrations at the dam of the Zelazny Most tailing pond varies in the range of 1–15 Hz. Generally, with lower energy and distance, higher frequencies are observed, while high-energy tremors in the far-field generate a seismic wave with relatively low dominant frequency. This fact was also proven by 6-DoF continuous measurement. As it may be noticed, the frequency content of translational seismic wave reached values from 0.15 up to 15.14 Hz. In turn, when examining the frequency of rotational seismic velocity, one may observe that dominant frequency of subsequent tremors is significantly higher in comparison to translational motion. In this case, observed frequencies varied in the range of 1.2 to 13.3 HZ (Figure 10). At the same time, the dominant frequency of seismic wave observed near the R-I shaft was significantly higher and reached almost 29 Hz in the case of rotational component and 45 Hz in the case of the translational wave (Figure 11). This was probably due to the proximity of the measuring site from the seismic source. The epicentral distance of 200–500 m was too small to efficiently filter high frequencies. One may conclude that in most cases, the dominant frequency of the rotational component was significantly higher than in the case of translational ones. Examining the frequency of the rotational component, it may be observed that in seventeen cases, the frequency of 12.8 Hz was clearly visible. Such a situation may suggest that this is an eigen-frequency of the concrete foundation on which the 6-DoF monitoring system was installed. Still, this topic requires further investigation.

The statistic of dominant frequency for each measuring direction at both monitoring stations is presented in Table 2. The measurement directions were marked as RX, RY, RZ in case of rotational velocity and TX, TY, TZ in case of translational components.

Analyzing the mean and median of dominant frequency distribution, it may be observed that on average, the dominant frequency of the rotational component is about 2–3 times higher than translational ones, and it drops significantly with the rise of the distance from the source. Still, the real effect of frequency content on object stability requires further investigation.

### 3.3. Damping of Seismic Wave

Precise determination of damping factors with the use of only two measurement posts is hard to conduct, due to the stochastic nature of tremors in terms of their energy and location. Because of high geological variability, the attenuation factors of seismic waves differ significantly in the LSCB region. Therefore, precise and reliable determination of damping factors requires a well-developed seismic network, to cover the whole area of interest and maximize the accuracy of the analysis. Lack of measurements with multiple stations located in one straight line with seismic wave propagation makes the calculations of damping factors inaccurate [54]. Still, it is possible to compare in a qualitative way how rotational and translational components of seismic wave are damped, by analyzing the values of *PG_V_* and *PH_R_* generated by the same events at different sites.

**Figure 12:**
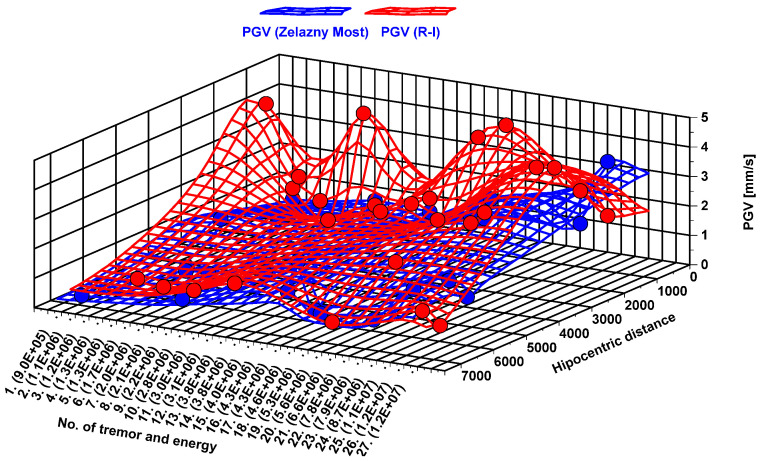
Comparison of PG_V_ recorded at Zelazny Most (blue) and near the Rudna-I shaft (red).

Therefore, 27 seismic events with the known location of their hypocenter were used to determine the difference in damping between rotational and translational seismic movement. As it may be observed from the three-dimensional (3D) surface map (Figure 12), the difference between *PG_V_* at the Zelazny Most dam and near the Rudna-I shaft differs up to 15 times. On average, the difference in recorded seismic velocities was about 2.5 times. The greatest disproportion in recorded *PG_V_* values (15.4 times) was observed in the case of a seismic tremor with energy 1.1 × 10^6^ J located 1.2 km from the Rudna-I shaft and 6900 from Zelazny Most, where *PG_V_* values reached 4.01 and 0.26 mm/s, respectively. In turn, the smallest difference (1.05 times) was observed in the case of tremors located at equal distance from both measuring sites, like a seismic event with the energy of 2.8 × 10^6^ J located ~6.8 km away from both 6-DoF measuring sites. In this case, *PG_V_* values were 0.9 in the case of the R-I shaft and 0.95 at the dam of the tailing pond. 

**Figure 13:**
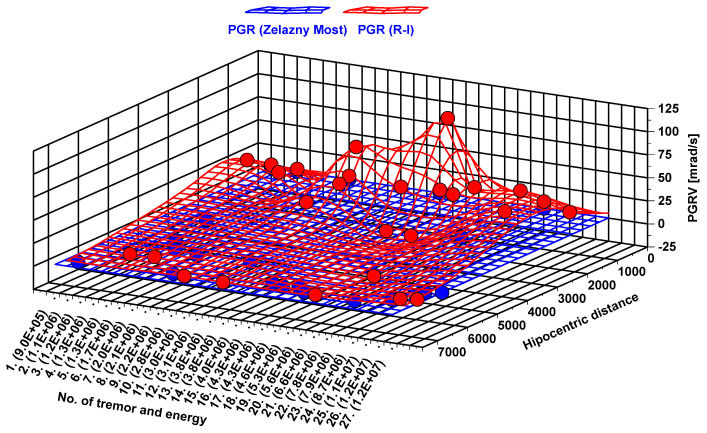
Comparison of PGRV recorded at Zelazny Most (blue) and near the Rudna-I shaft (red).

At the same time, damping of the rotational seismic wave generated by exactly the same tremors differs significantly from translational ones. Namely, with the rise of distance, recorded values of rotational velocity drop considerably. As a result, *PG_RV_* recorded at the slope of the Zelazny Most dam, in most cases, was a few thousand times smaller than in the case of measurements near the Runda-I Shaft (Figure 13). The biggest difference of rotational values at measuring posts exceeded 2500 times and was observed as a result of a seismic tremor with the energy of 7.9 × 10^6^ J. Hypocentral distance of this tremor was 1 km from the Runda-I Shaft and 6.8 km from tailing pond dams. 

This is a clear premise that in LSCB conditions, the rotational component of an induced seismic wave is significantly more damped that the translational one. Therefore, full recognition of the seismic effect on adjacent structures should be based on data obtained from 6-DOF measurement, especially in the near-wave field. 

### 3.4. Empirical Formulas for Rotation Prediction for Both Measuring Sites

The lack of a 6-DOF monitoring system determines the fact that currently, there are no developed equations for rotational velocity prediction in the LSCB area. On the basis of gathered data, the predictive formulas for peak rotational velocity assessment both for triaxial (*PG_RV_*) and horizontal (*PH_RV_*) measurements may be developed. 

Recent formulas for seismic velocity predictions in the LSCB are based on a linear relationship according to the formula:

(5) $$PG_{RV}=a\cdot R_{E-d}-b$$

where: *a*, *b*—constants describing slope of the linear dependence, while *R_E–D_*—reduced distance, m/J. 

The parameter called reduced distance, *R_E–D_*, allows to define the effects of both distance and energy of the tremor on the value of peak velocity near the measuring site. The *R_E–D_* may be calculated according to dependence:

(6) $$R_{E–D}=\frac{Log_{10}{\left(E\right)}^{\alpha }}{L^{\beta }}$$

where: *E*—energy of seismic event, J, *α*—energy-scaling factor determined on the basis of field measurements, *L*—distance from the seismic event, m, *β*—distance-scaling factor determined on the basis of field measurements.

Factors *α* and *β* are strictly related to the local geologic condition, thus accuracy of reduced distance calculations are strictly related to the quantity and quality of field-gathered data. 

Based on tremors database and field measurements, the constants α and β were determined. The reduced distance was defined in an iterative way, separately for both measuring sites. Successive iterations were performed until the maximum correlation between the *R_E–D_* value and the calculated *PG_RV_* and *PH_RV_* were obtained. It was assumed that the relationship between tremors observed in the near-field and recorder values of rotational velocity should be linear, so correlation was analyzed with the use of Pearson’s correlation coefficient. 

By definition, the correlation between the variables *X* and *Y* is a measure of the strength of the linear relationship between analyzed variables. Pearson’s correlation coefficient is determined from the formula:

(7) $$r_{xy}=\frac{\sum \left[\left(X_{i}-\bar{X}\right)\cdot \left(Y_{i}-\bar{Y}\right)\right]}{\sqrt{\left[\sum {\left(X_{i}-\bar{X}\right)}^{2}\right]\left[\sum {\left(Y_{i}-\bar{Y}\right)}^{2}\right]}}=\frac{\frac{1}{n}\sum \left(X_{i}Y_{i}-\bar{X}\bar{Y}\right)}{\sigma_{X}\cdot \sigma_{Y}}$$

where: $X_{i}$, $Y_{i}$—*i*—value of the *X* and *Y* dataset, $\bar{X}$, $\bar{Y}$—average of *X* and *Y* population, $\sigma_{x}$, $\sigma_{y}$—standard deviation of *X i Y*, and n—number of observations.

**Figure 14:**
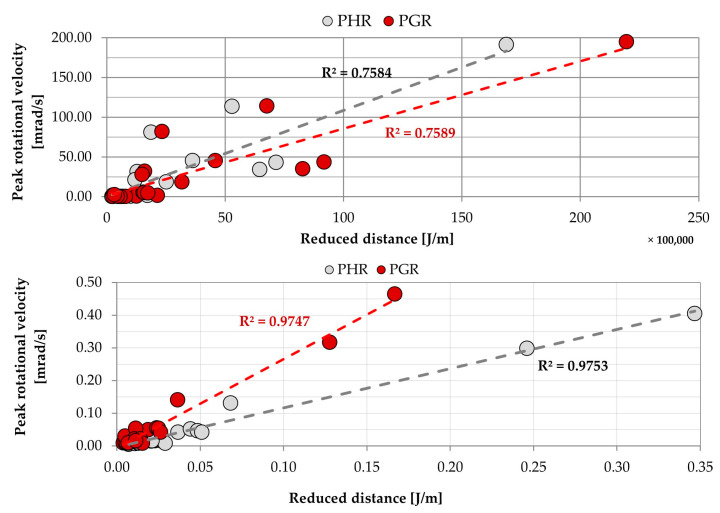
The relation between R_E–D_ and recorded rotational velocity at the Rudna-I measuring site (**top**) and Zelazny Most (**bottom**).

In Figure 14, graphs representing the dependence of peak rotational velocity, both in horizontal and triaxial terms, and reduced distance are shown.

Results obtained at site number 1 are scattered significantly around the trend lines, but a clear dependence between peak values of rotational velocity and reduced distance may be observed. Calculated coefficient of determination exceeded the value of 0.75 both in the case of horizontal and triaxial calculations. At the same time, the dispersion of values observed at the Zelazny Most dam was visibly lower. As a result, calculated coefficients of determination exceeded the value of 0.97, which represents a well-fitted model. The constants and results of calculations are presented in Table 3.

Finally, predictive equations for peak ground rotational velocity and peak horizontal rotational velocity in the near-wave field were developed for both test sites. The new formulas are as follows:

(8) $$PG_{RV\;\left(Zelazny\;Most\right)}=1.389079343\cdot \left(\frac{\log_{10}(E^{7.8953})}{L^{2.1367}}\right)-0.0074$$

(9) $$PH_{RV\;\left(Zelazny\;Most\right)}=2.070050389\cdot \left(\frac{\log_{10}(E^{7.5317})}{L^{2.1311}}\right)-0.0037$$

(10) $$PG_{RV\;\left(Rudna\right)}=0.00000883232\cdot \left(\frac{\log_{10}(E^{12.92897953929})}{L^{1.25865}}\right)-2.62698106401025$$

(11) $$PH_{RV\;\left(Rudna\right)}=0.0000087128\cdot \left(\frac{\log_{10}(E^{12.70816394})}{L^{1.23153}}\right)-1.925186565$$

**Figure 15:**
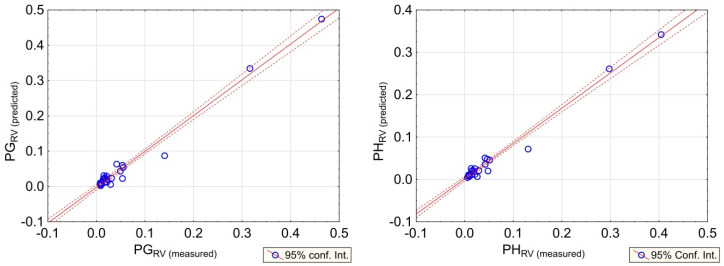
Regression plots for predicted vs. observed values of PG_RV_-**left**, and PH_RV_-**right**, at the Zelazny Most tailing pond.

**Figure 16:**
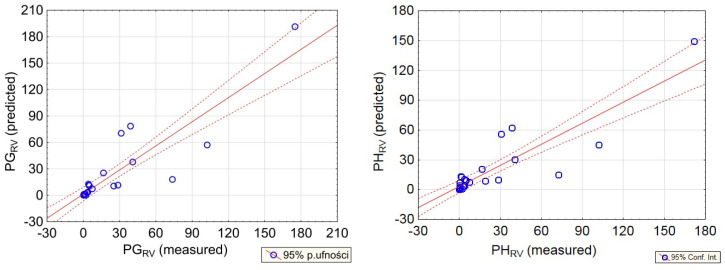
Regression plots for predicted vs. observed values of PGRV-**left**, and PHRV-**right**, at the Rudna-I shaft.

To determine the reliability of prediction based on the new formulas, the regression plots of predicted and measured values of rotation velocity were prepared (Figure 15 and Figure 16). When examining the results obtained at the Zelazny Most dam, high reliability of the obtained model may be observed. The generated error of prediction did not exceed 30% in any of the cases. Therefore, the accuracy of prediction is satisfied. 

In turn, the accuracy of seismic rotational velocity prediction at the Rudna-I site was considerably lower. This is mainly due to the very close vicinity from the source of vibration, and significant changes of direction of seismic wave propagation. Moreover, there are many factors which may significantly affect the level of ground vibration, and which were not determined in this analysis. One of them is the detailed description of the geology and mining conditions at the path of each seismic wave generated by mining tremors [55,56].

## 4. Discussion

According to past studies, the highest peak rotational velocities recorded so far do not exceed dozens of mrad/s [17,57,58]. Therefore, data gathered during one year of measurement in the LSCB region are of great value because collected waveforms were characterized by high rotation rates. Depending on distance from mining tremor and its energy, the recorded rotational velocity varied from a few μrad/s up to 190 mrad/s, which is five times more than in the case of measurements of rotation generated by blasting of 1 kt of explosives in underground conditions from a distance of 1 km [57]. As it was pointed out in many studies [17,59,60,61,62], there is a strong correlation between the logarithm of peak ground rotational velocity and peak ground translational acceleration. Our research indicated that this is true but only at higher distances from the high-energy seismic source, where rotation is significantly damped. At very close distances, not exceeding 2 km in hypocentric terms and 500 m in the epicentric domain, the rotational velocity does not correlate with translational acceleration. Moreover, it may be noticed that the rotational component of ground motion wave is characterized by much higher dominant frequency and attenuation in comparison to the translational one. This suggests that the most significant impact of this type of seismic movement needs to be monitored and analyzed, especially in the near-field where expected rotational velocity may reach even up to a few hundred mrad/s in case of high-energy tremors. Taking the above into consideration, it may be stated that characteristics of seismic wave propagation in near- and far-field differ significantly in the case of the rotational component of seismic waves. At a close distance, the rotational effect generated by wave propagation does not correlate with translational ones. In turn, at greater distances, the rotational component is significantly damped and strong dependence between translational and rotational components may be observed [55,56,57,58]. Therefore, there is a necessity of separately determining a predictive formula for the near- and far-wave field seismic events.

Concerning the LGCB area, the measurement of rotational seismic motion was, until now, totally neglected. Therefore, there have been no attempts in the past to develop empirical relationships describing the relation between the energy of mining tremors, their hypocentral location, and values of rotational velocity or rotation rate. Our database considered 29 high-energy events in the case of the Rudna-I shaft and 38 in the case of the Zelazny Most tailing pond, respectively. Such population of results on the one hand allows to conduct reliable prediction, which was proven by regression analysis, but still, on the other hand, there is a possibility of further improvement of calculation accuracy, with the expansion of the high-energy tremor database. 

The rotational components of seismic waves, despite very extensive research studies, are still a topic which needs to be investigated in detail, especially in the areas of mining-induced seismic activity, where the possibility of the occurrence of a seismic event in the near wave-field is relatively high. Further measurement of 6-DoF seismic motion at the different types of structures will allow for determining how they are affected by rocking motion and tilt. In the case of the LGCB area, the gathered results will be useful, for example, for the purposes of stability analysis of the Tailing Storage Facility Zelazny Most, which in a few years will be the biggest object of its kind in the world. Implementation of additional 3-DoF into stability calculation has not been investigated so far, and thus the analysis of the real effect of seismic rotation on geotechnical object stability is an important issue. 

## 5. Conclusions

Within this paper, the results of eleven months of 6-DoF measurement in the Lower Silesian Copper Basin, Poland, were presented. Measurements were conducted with the use of two sets for joint monitoring of rotational and translational seismic motions manufactured by Eentec company. Both sets were equipped with a rotational seismometer R-1, a translational seismometer EP-300, and a portable recorder DR-4050P. In the analysis, only high-energy tremors in the near-field were considered. Low-energy tremors (E < 1.0 × 10^6^) were omitted. The significant differences in terms of damping and frequency distribution were found. In general, the frequency of rotational motion was on average 1.5–2 times higher compared to translational motion. The detailed description of attenuation requires further in-field measurements with the use of a higher number of seismic stations, to determine the effect of local geologic condition on rotational velocity damping rate. In terms of amplitude, the maximum recorded rotational velocity reached a value of 195 mrad/s, which was related withrotation rate at the level of 0.45°. From the object stability point of view, it may be expected that such rotation of, for example, a tall shaft fundament or slopes of a high earth dam, may significantly affect its stability.

The developed predictive equation allowed us to assess the rotation rate near the measuring site with acceptable reliability. The results will be also be a basis to continue research aimed at the mathematical description of the influence of rotational motion on object stability. 

## Figures and Tables

**Table 1:** Basic parameters of seismometers utilized for preliminary 6-DoF measurements.

Parameter	EP-300 (Translational)	R-1 (Rotational)
Operating principle:	Electrochemical motion transducer with force-balancing feedback	Electrochemical motion transducer
Dynamic Range:	150 dB @ 1 Hz	110 dB
Bandwidth:	0.0167–50 Hz	0.05 to 20 Hz ± 3 dB
Power Standard:	10–15 VDC	9 to 14 VDC
Supply current:	30 mA	20 mA

**Table 2:** Descriptive statistics of dominant frequency distribution at both measuring posts.

	Zelazny Most						Rudna-I					
	RX	RY	RZ	TX	TY	TZ	RX	RY	RZ	TX	TY	TZ
**Mean**	4.87	8.05	4.25	2.37	2.74	2.71	10.70	10.51	9.92	4.04	7.03	7.03
**Median**	4.18	8.24	3.86	1.50	2.08	1.12	12.77	11.13	12.76	2.30	3.34	4.76
**Minimum**	1.46	2.28	1.20	0.20	0.27	0.15	0.65	3.24	0.26	0.15	0.62	0.62
**Maximum**	13.30	12.84	9.30	12.76	12.80	15.14	25.59	23.91	28.69	12.90	45.61	23.91
**Standard deviation**	3.00	2.35	2.06	2.53	2.63	3.31	4.87	5.76	5.46	3.65	10.21	6.48

**Table 3:** Coefficients of correlation and empirical parameters for rotation prediction formulas.

Parameter	Zelazny Most	Rudna-I
**Peak Ground Rotational Velocity (*PG_RV_*)**		
Coefficient of determination ***R*^2^**	0.9747	0.7584
Pearson’s correlation coefficient ***r_xy_***	0.9872	0.8708
Energy scaling constant α	7.89530	12.9289
Distance scaling constant β	2.1367	1.2586
**Peak Horizontal Rotational Velocity (*PH_RV_*)**		
Coefficient of determination ***R*^2^**	0.9769	0.7589
Pearson’s correlation coefficient ***r_xy_***	0.9884	0.8711
Energy scaling constant α	7.5317	12.7081
Distance scaling constant β	2.1311	1.2337
